# Atlanta Society of Medicine

**Published:** 1893-06

**Authors:** 


### *SOCIETY REPORTS.*

### ATLANTA SOCIETY OF MEDICINE.

ATLANTA, GA., March 14th, 1893.

The regular order of the meeting was a consideration of the

#### TREATMENT OF CHRONIC GONORRHŒA.

DR. F. W. MCRAE opened the discussion. (See page 200 of this issue.)

THE PRESIDENT said: Gentlemen, you have heard Dr. McRae.

*With Jones' suspension speculum the dorsal position is best. This excellent though ponderous instrument allows the cervix to sink within easy reach, when it may be held and studied with the spread blades of a dilator between which he may do all this work and inject if he wish the styptic antiseptic. It promptly controls the hemorrhage if he has done the previous work well.

This is a regular discusssion and I will now call upon the members of the society.

DR. DUNCAN said: These are very troublesome cases. They worry every physician who has anything to do with them. We all know that they have given us a great deal of trouble heretofore, and we expect them to give us trouble in the future. I am glad to know that Dr. McRae has been so successful with his plan of treatment. I have tried several plans, and in the main have been successful with the few cases I have had to treat, but they worried my patience very much.

I have tried disinfectants of a weaker nature, for instance, boracic acid solution (saturated solution). I have an instrument I got for the purpose which had a shoulder to it and with the eye opening backwards. After the patient had urinated, I would introduce that beyond the point where I thought the sore was and flush out the urethra, using large quantities of water. I got good results from that. It was very beneficial in cleansing the part. Then I made a stronger application of remedies to the mucous membrane. But, I have not had a great deal of experience with this trouble and am not making a specialty of it.

DR. STOCKARD said: I just came in to-night and did not intend to make any remarks. I have treated a good many cases of this trouble, being a practitioner in rather a small place, and we have to treat almost everything that comes about us. I have been, for years, treating them with injections such as Dr. McRae described, with a long nozzle syringe, having used most of the time a Bumpstead syringe with a number of perforations in one end. For the last two years I have been using Keyes' modification of Ultzman's syringe, and have had very good success in most cases.

I was struck several months ago by this fact: A patient I had been treating for some time went to New York and consulted Dr. Keyes. When he came back he brought a letter from Dr. Keyes advising the continuation of the injections of nitrate of silver, very much the same as I had been using. After giving him an injection, he said, "This does not feel like some of the injections Dr. Keyes gave me." Although he began with one grain to the ounce, I had been using a much stronger solution. He said the injections

Dr. Keyes gave him produced a strong desire to urinate and kept it up for half an hour, whereas the injections I gave him did not keep it up. He persisted that it must be due to the syringe, so I got a Tieman catalogue and found that the syringe Dr. Keyes had been using had the opening in the end. After I got one of them, I found I could produce the desire to urinate too. He said Dr. Keyes considered that sensation a test of the efficacy of the treatment. After using it awhile I did not find that there was anything in it. I did not find that I could cure any greater number of cases. Possibly, when the trouble is beyond the cut off muscle, the other syringe does not throw the injection back far enough, but outside of that, I do not know that there is any difference. I do not know anything more I can say on this subject. We have all had about the same experienee I expect. We find some troublesome cases and a few that we cannot cure no matter what we do.

Dr. Hurt said: Mr. President, I do not know that I can improve on the treatment already suggested by Dr. McRae. As to the experience I have had in the treatment of this trouble, I am glad to say that I have had very few cases that have been as obstinate as some I have seen reported somewhere, but I am sorry I had that many. They worried me a great deal, one case especially. He was a small man and very small in all his parts. I have seen him when he seemed to be shriveled up to nothing. Yet, he had his gonorrhœa and it stayed with him and defied all the efforts I could make to eliminate it from him. I think I treated him over a year and left him uncured. I believe, had I pursued the course suggested by Dr. McRae, I might have relieved him. However, in the latter few years of my experience, I have adopted a treatment something of this kind: the introduction of gum elastic bougies, followed by the injection of some mild solution, not so frequently nitrate of silver as a mixture somewhat after this formula: say two drachms of boro-glyceride, two drachms of colorless solution of hydrastis and bismuth and I sometimes incorporated with that two drachms of f. e. hamamelis, adding water enough to make two ounces. I used the bougie every two or three days, according to the irritation it may have excited, and used the injection every four hours to once per day, according to indications. Now,

I look upon boro-glyceride as being antiseptic and somewhat astringent. The pressure of the instrument, when passed in, was upon the purplish and engorged mucous membrane of the urethra, and in that way, it is somewhat depleting. Follow this by the mild injection, and, by the way, this injection is held in the urethra a sufficient time, and by a little pressure of the finger and thumb it is forced as far as may be and held there one to two minutes. Under that treatment I have had good success in managing some obstinate cases. I am rather doubtful whether it would be succeesful in some of the more severe cases, still I think it well worth the effort if these other means fail.

It ought to be the purpose of the profession, and doubtless is, to give as little pain as possible in the treatment of any disease, but, of late years, we rather employ all antiseptics, which are unable to get hold of these things. This is not an original idea with me and I do not claim that it is by any means specific, but I have found a number of cases who, as Dr. McRae says, have expressed themselves as feeling decidedly more comfortable after the introduction of a bougie, followed by this injection. In some cases I direct that it be used every four hours during the day, and in others I direct its use only once per day. I have required my patients to come to my office and let me make the injection. I did this in order to more carefully watch the effects, and I have been very much gratified in most instances at the relief of the patient. I, however, believe that nitrate of silver in weak solution is an effective means of relieving it and would not discard it at all. I would use it in those cases I found difficulty in curing with this milder solution.

I have not had any very obstinate cases, therefore I expect my experience with troublesome cases is rather more limited than Dr. McRae's.

Now, as to internal treatment: I sometimes keep the patient on cubebs or sandal-wood, either of which, I think, is very effective in aiding the other treatment. In fact, I always combine it, and in some cases I am not so sure that I have not left it alone when I thought it was very much needed, and subsequently taken up the use of it.

DR. AVARY said: I am sorry, Mr. President, I did not hear all of that paper by Dr. McRae. I have not treated a case of gonorrhœa in the male for something like five years, and never used nitrate of silver in my life. Recently, I have had my attention directed, as most other doctors have, to gonorrhœa in the female, supposed to have been caused from chronic gonorrhœa in the male. I do not know whether Dr. McRae said how long the gonococcus might be able to propagate its race in the male urethra or not.

(Dr. McRae: I did not say, but it is my observation that they are capable of doing so during the natural life of the man, in some instances.)

I have had, I think, some cases of gonorrhœa in the female where no history of any contagion was likely to have taken place, unless it had been from a very old case of gonorrhœa in the husband. I have had, of course, the usual run of gonorrhœa in the female, or pyosalpinx, which some claim cannot be established except by gonorrhœa. I have no special opinion on the subject.

I will state, however, that I thought I saw good results from frequent irrigation, not so deep, in acute cases, with mild solutions of bichloride of mercury and cocaine, about the time I ceased to treat it. I gave as much cocaine as the patient was able to pay for and a 1-2000 solution of bichloride of mercury, and directed it to be used as often as possible during the day, as frequently as every hour, if pus showed itself at the meatus.

DR. KIME said: As for my experience in the treatment of gonorrhœa in the male, I have nothing to say on that line. I can only emphasize the fact that I feel the physician is responsible for the sins of omission as well as commission. In other words, I think a great many physicians are responsible for the serious results of gonorrhœa from the fact that they fail to caution their patients of the tendency of gonorrhœa to exist so long in the male uncured. They fail to caution them of the evil results of sexual intercourse until they are satisfied beyond the peradventure of a doubt that gonorrhœal inflammation has been entirely cured in the male.

It seems to me that so long as physicians treat gonorrhœa in the male so lightly, then it is that those who are following in the line of gynecological work will continue to find such serious results in the female. I feel that any physician who treats a case of gonorrhœa in the male and fails to warn him of the danger has failed to do his duty as a man in his profession, and that he is, in a measure, responsible for the results occurring from that man having sexual intercourse which develops trouble in the female.

Dr. Childs said: I have had some experience in treating chronic gonorrhœa and have never had any disease to treat that gave me as much trouble. I have tried almost everything in some cases, and in one I think I failed altogether to cure the man.

Otis claims that most cases of chronic gonorrhœa are due to stricture. He calls every narrowing of the urethral canal a stricture. I think he calls a normal narrowing a stricture. Lydston claims that a great many of these cases which Otis calls stricture are not strictures, and that the inflammation which continues there is due to a localized infection back of one of these narrowings.

Otis's results are due to the reduction of these narrowings, and making application to that inflamed spot. I have tried passing sounds until I dilated the urethra to its full capacity without success. I have tried irrigation with bichloride of mercury, as hot as the patient could bear it, not as strong as Dr. Avary used, about 1-3000, and have gotten fairly good results from that. At first I used the solid steel bladed instrument, but found that that did, if anything, more harm than good. Every time it was passed in, instead of relieving the patient, it made matters worse. I then commenced using the soft rubber catheter for irrigation and found I got good results.

For the last few months I have been using an instrument like that shown by Dr. McRae, and can say that it has given me better satisfaction than anything I have ever used. I have used it in one case of acute gonorrhœa. I irrigated the urethra and then applied nitrate of silver, 1-4000. The next day the discharge had ceased almost entirely, and now there is scarcely any at all. This is the only acute case of gonorrhœa in which I have used it.

Another plan from which I have gotten fairly good results is a mixture of sub-nitrate of bismuth and water injected into the urethra. That seems to keep the two surfaces apart and gives good results but of all the instruments I have used, the soft rubber catheter, with nitrate of silver solution, gives the best results.

One thing I do, which Dr. McRae does not do, is to irrigate the urethra before applying it, so as to get rid of the sodium chloride in the urethra, which precipitates the nitrate of silver. By not irrigating the urethra I do not think you get the results that you would get by irrigating it. Have the patient pass his urine and then irrigate the urethra with hot boracic acid solution, as hot as the patient can bear it, perhaps 105° to 115°.

The urethra seems to bear hot water equally as well as the female vagina. Then inject your nitrate of silver solution and it gives good results.

DR. CROW said: I have never had any experience of any kind in the treatment of gonorrhœa in the male. During the seven years I have been here I do not suppose a half dozen cases have come under my observation. I make it a rule to refer them to other gentlemen.

But I have felt a deep interest, directly or indirectly, in the results of this terrible malady which we are discussing. It was my good fortune last fall in New York to hear Prof. Gouley, who is authority on genito-urinary troubles, deliver a lecture on chronic cystitis and allied renal affections. He presented to the class at that time a number of specimens, showing the result of gonorrhœa, extending to the ureters and kidneys. It passes up to the ureters, producing inflammation there, and on up to the kidneys, producing many terrible diseases with which we are all acquainted.

This subject is dwelt upon by men who are studying diseases of women.

Blanton Sutton, an English author, who has just gotten out a work on the ovaries and fallopian tubes, if I remember aright, attributes salpingitis and ovaritis, to a large extent, to infection from gonorrhœa. So also does Martin, a German author, whose work is translated by Cushing, of Boston, and the same thing is spoken of by Pozzi, a French author. Some of these gentlemen claim that latent gonorrhœa will develop an acute inflammatory trouble of an infectious nature, the gonorrhœa having been latent for a number of years. For instance, a young man at fourteen will have several cases of gonorrhœa, which pass back and involve the deep urethra, and possibly he may have some localized cystitis. This will pass off and probably he will marry in six or eight years. After that, under favorable circumstances, that man may infect his wife. If she has a vaginitis from any cause it is possibly from this latent gonorrhœa. I suppose there is some gleet remaining. It may develop cystitis, or it may go on to a salpingitis or pyosalpinx.

Last spring I operated on a case of abscess of the ovary, specimen of which I showed to the society, which I attributed to infection from gonorrhœa, and I suppose every man who has done any work in this line has had cases which were very clear to his mind as to their origin. All authorities agree that it is the most prolific source of infection. I know practically nothing of its treatment in the male, consequently I will say nothing about it.

DR. HUTCHINS said: Mr. President, the whole subject of chronic gonorrhœa gets down to the practical question as to the situation of gonococcus. If the gonococcus is not deeply situated, of course it can be more readily reached and destroyed, and the treatment of such cases would be correspondingly simple. If the gonococcus, as has been claimed, has penetrated very deeply into the mucous membrane, or if it has gone into the little openings into the urethra (I do not remember the names of them just now), the difficulty is not what you shall use, but it is whether you can get your application down to the gonococcus and destroy it. Of course, to do that you have to use the proper method of injection and bring the fluids in contact with the gonococcus in order to destroy it. If you can do that, that is the end of the case.

I was reminded of a very terrible case I read of a year or two ago in the *New York Journal of Cutaneous and Genito-Urinary Diseases*. It was the case of a young man who had gonorrhœa, who had postponed his marriage a time or two on his physician's advice. Finally, thinking that he was well enough and there was no danger, he married. The result was a most terrible case of vulvo-vaginitis and everything else in the neighborhood diseased, and the young woman came very near dying. It was a horrible case, as described, and yet the young man seemed well.

DR. ELKIN said: I did not intend to say anything at all on this subject, though it is one of considerable interest to me. I have had a good deal of experience in the treatment of venereal diseases, a good deal of experience in the treatment of gonorrhœa and a good deal of troublesome experience in the treatment of chronic gonorrhœa or gleet.

I have used the treatment spoken of by Dr. McRae, and have found it very beneficial in a great many cases. In some cases, though, I have failed to get the beneficial effect from it, but I think probably that fact was due as much to the patient as it was to the plan of treatment. That has been the great trouble I have found with these patients, anyway, to get them to carry out systematically any plan of treatment. If they do not carry out the instructions with reference to diet, exercise, drinking of stimulants, they do not get the beneficial effects from the treatment, no matter what plan you adopt. But, for deep urethral irrigation, I am satisfied we get more benefit from the plan just mentioned of injecting the deep urethra with solution of nitrate of silver than any other. I have done that very frequently lately and gotten marked effects. I have now under observation a patient I treated for chronic gonorrhœa. Every time I give him an injection he feels that he is perfectly well and does not come back any more for two or three weeks. Then he comes back for another injection. I think he would get well if he would be more systematic. He was also treated by Dr. Keyes, of New York, who also used this treatment with the silver catheter. This is what I use if there is not much inflammation. Where the mucous membrane is very sore I use a soft rubber catheter. I generally irrigate the urethra first with a solution of boracic acid in warm water, and then make my application.

I think, however, we would get less chronic gonorrhœa if we were more careful in our treatment of the acute cases. A great many cases become chronic as a result of neglect, either through ignorance or carelessness on the part of the physician, or else carelessness on the part of the patient in carrying out the instructions of the physicion who knows how to treat this condition. This has been verified in a great many instances. I have had many cases of gonorrhœa in the subacute stage come to me from highly reputable physicians who never would examine them. They would write out a stereotyped prescription and tell the patient to inject it two or three times per day. Now, there is a great deal of technique in the mode of treatment, which ought to be carefully explained to the patient. The patient ought to be instructed how to inject and as to the kind of syringe to get. I think that has, in the majority of cases, a great deal more to do with the effect you get than what you give or use. I think probably other medicines would have as beneficial an effect on the deep urethra, although I have not used them, but it is just the way of giving them.

I indorse heartily all Dr. McRae has said, and I have used that treatment. In the anterior portion of the urethra I frequently use nitrate of silver as an injection with an ordinary syringe.

DR. WESTMORELAND said: My experience with chronic gonorrhœa is that, after the cessation of the gonococci, which are generally connected with the epithelium, which is thrown off during the primary stages, as a rule, that discharge can better be cured by cutting the meatus than it can by injections. Every case of that kind I have, if it continues for a long time, if there is any stricture of the meatus, I simply cut it to the full size, and after I think the gonorrhœa has subsided, I begin to use sounds into that portion of the urethra which is affected. Where the disease has gone into the bladder, of course you cannot afford to use sounds in this stage down to the bladder. I use sounds in these cases for two reasons. One reason is that, as Dr. McRae has mentioned, these little ducts empty into the urethra all the way, and these openings always become more or less affected, and I think the infection in the chronic stage is rather in them than in the urethra proper. If you use in this case a very large sound, you accomplish the very thing Dr. McRae spoke of with reference to the seminal vesicles. By this pressure you press out all the secretion in them. This secretion is not washed out by the urine, for they open in the same direction that the urine flows. In using sounds I do so without fear of infection after that length of time—I mean after the elimination of the gonococci to a certain extent. I take it for granted that the urethra in these cases has become more or less accustomed to them, and they do not have that power of infection. In using the sound in these cases I do not stop by simply putting it in. I let it stay there three, four or five minutes. In this way you accomplish pressure, and at the same time get a more or less stimulating effect from the cold steel bougie.

At the same time I think the usual remedies which you give to correct acidity of the urine will do some good by preventing more or less irritation which you would otherwise have. Where you have the recurrent attacks that the doctor spoke of, I have found that they can be corrected by cutting the meatus where they cannot be corrected by the simple use of injections. In fact I have found that where you use injections for too long a time, the urethra gets accustomed to that stimulation just in the same way as a man does who takes five or six drinks per day, but when you take away that injection, no matter what solution you use, you will be apt to have a recurrence of that discharge, particularly if your man happens to get "out on the town" and take a few drinks and spend the night with a woman. On the other hand sometimes that itself will cure the patient. As an illustration of it, I had a very peculiar case, in which I had used all the injections I knew of. As long as I kept the patient in bed and kept him quiet, he had no discharge. I was using the injections three times per day. If he would get up and take a ride or walk, however, when he would get back the discharge would be about the same it was before he started. As a *dernier ressort* I told the patient I did not know what to do for him, but that I had a suggestion to make, to the effect that he go to a dance, drink all the champagne he could, dance all night, or the most of it, and then spend the balance of it with some lady of easy virtue, and if that did not cure him he had better get another doctor. He followed out that plan of treatment exactly—I do not know but what he went beyond instructions a little—and he has had no discharge since then.

Sometimes revulsion in the manner of treatment will accomplish what we wish to accomplish by the regular old style treatment. So far as I am concerned, I do not believe in injections to any great extent, and in those cases where they have that persistent chronic discharge, I generally cut the meatus and depend more upon the introduction of sounds than upon injections.

In closing the discussion Dr. McRae said: I wish in the beginning of my remarks to indorse heartily what our President has said about the treatment of acute gonorrhœa. My experience is that a large number of the cases of gonorrhœa I get to treat are either subacute or chronic. They have been in the hands of physicians—some of them very reputable ones—who, as Dr. Elkin has said, have paid too little attention to this disease. That is the reason I have brought this discussion before the meeting to-night. It is simply because the practitioner does not realize his responsibility in the treatment of this terrible disease. If I had to select which I had rather have, between gonorrhœa and syphilis, it would take me a long time to decide.

A large proportion of the cases are treated by the druggist. A young man contracts gonorrhœa, and he does not want to go to a physician. They all know some nice young man who is a druggist and with whom they associate, and they usually go to the druggist. As a result, if he does not compound something for them, he will tell them that he has a number of patent medicines warranted to cure in from one to five days. That has not been my experience with gonorrhœa. Dr. Bryson, of St. Louis, one of the best genito-urinary authorities in this country, makes the statement that he believes no case is absolutely cured inside of a year. I think that is taking an extreme view of it, but it shows how men of large experience think about this disease.

As to the disastrous results following neglect of proper caution on the part of physicians, the case which Dr. Hutchins mentions—which was a report from Dr. Brewer, of New York, a man who is thoroughly posted in this line of diseases—is one of the many with which our journals are constantly filled. Dr. Price, of Philadelphia, who is in the front ranks on abdominal surgery in this country, and perhaps the most eminent in America, claims that a very large proportion of those cases of pyosalpinx (pus in the pelvis, as he calls it) are due to gonorrhœa, and almost all of these cases he can trace back to the time of infection.

Now, as to Dr. Childs, I fully indorse what he has said in quoting Dr. Lydston and Dr. Otis in reference to the introduction of bougies. I remember one case—a medical student. I treated him through two full courses of lectures for chronic gonorrhœa. He had it when he came here, and when he left he took it with him. That was four or five years ago. I do not know whether he has it now or not, but I can testify to the fact that he had it when I got through with him.

Now, as to the irrigation of the urethra. That, of course, allows you to use a weaker solution with greater effect than you could without irrigation. If you do not use irrigation beforehand, all that is necessary is to use a little stronger solution, and then I use it in the strength of all the way from 1–6000 to 1–3000 or 1–2500. I have not used greater strength than that in deep injections.

As to the frequency of the injections, I find my patients do better to give them an injection every third day. I had one patient recently who was so much benefited by it that he called every day for me to give him an injection. I treated that patient for several weeks. He was one of the most intractable cases I ever had, because I could not keep him from drinking and from indulging in sexual intercourse. He regularly told me all the time I was treating him that he indulged in venery three or four times per week. He was finally cured. I treated him about three months, using the injections every third day.

There is one thing in some cases which will complicate matters very much, and that is extension of this inflammation in the seminal vesicles. Occasionally gonorrhœal inflammation will find its way through direct continuity of surface into the seminal vesicles. There it becomes localized, and I know of no method by which it may be dislodged from its position. It may be diagnosed by first having the patient urinate and then introduce your finger into the rectum and milk the base of the bladder. If they are much inflamed you will feel them. In that way you will milk the secretion out into the urethra. Then have the patient urinate again and you will find this discharge in the urine, where it may be examined by a miscroscope to see if there is pus in it.

The length of time required to cure this disease is very variable. Some of them may be cured, as one case reported to-night was, by one or two injections, and others may require months of treatment.

I do not believe there is any case of chronic gonorrhœa which ought not to be cured if it is confined to the urethra or neck of the bladder. I believe all these cases can be cured. We cannot cure them unless we get the control of the patient though. That is one essential in the treatment. Unless we can get absolute control of the patient, it is better for ourselves, as well as for the patient, if we have nothing to with them. Cases cannot be cured without this control.

Now, one of the reasons why this is the best method of treatment, is the location of the disease, which Dr. Hutchins has mentioned. The gonococci bury themselves deeply into the urethra and into the follicles which are given off. These lacunæ are found in the urethra throughout its extent, and the disease becomes localized in these follicles. By this method of treatment, and from the distending of the urethra, and more or less distending the follicles, you get a better effect from this injection.

Now, this is all I shall say in closing this discussion. I wish to impress again the importance of the disease, and the great trouble about it is, that everybody is considered competent to treat either the acute or the chronic form, and most physicians pay no attention to it whatever. They think it makes no difference if they do not cure it; it will run on three or four or six weeks and the case will get well; that when the discharge ceases he is well. A physician in Atlanta told me not many months ago, in speaking of the treatment of this trouble, that he cured all his cases in two to three weeks. I said, "Doctor, I wish I knew how to cure all my cases in that length of time. I can stop the discharge to a great extent in that length of time in a certain number of cases, but after leaving off the treatment they come back to me or go to some one else with another case of inflammation." "Well," he said, "I simply tell them they have caught another case, and charge them another fee."

